# Cell Suspension Culture of *Eriobotrya japonica* Regulates the Diabetic and Hyperlipidemic Signs of High-Fat-Fed Mice

**DOI:** 10.3390/molecules18032726

**Published:** 2013-03-01

**Authors:** Chun-Ching Shih, Jiun-Lin Ciou, Cheng-Hsiu Lin, Jin-Bin Wu, Hui-Ya Ho

**Affiliations:** 1 Graduate Institute of Pharmaceutical Science and Technology, College of Health Science, Central Taiwan University of Science and Technology, No.666, Buzih Road, Beitun District, Taichung 40601, Taiwan; 2 Department of Internal Medicine, Fong-Yuan Hospital, Department of Health, Executive Yuan, Fong-Yuan District, Taichung 42055, Taiwan; 3 Graduate Institute of Pharmaceutical Chemistry, China Medical University, Taichung 40402, Taiwan; 4 Jen Li Biotech Co. Ltd., Yong-Feng Road, Taiping District, Taichung 41143, Taiwan

**Keywords:** *Eriobotrya japonica*, terpenoids, diabetes, hyperlipidemia, AMP-activated protein kinase phosphorylation

## Abstract

The present study investigates the anti-hyperlipidemic and antihyperglycemic effects and mechanism in high-fat (HF)-fed mice of cell suspension culture of *Eriobotrya japonica* (TA), which contains a great number of pentacyclic terpenoids. Firstly, C57BL/6J mice were randomly divided into two groups: the control (CON) group was fed with a low-fat diet (n = 9), whereas the experimental group was fed a 45% HF diet for 8 weeks. Afterwards, the CON group was treated with vehicle, whereas the HF group was subdivided into five groups and was orally given TA or rosiglitazone or not for 4 weeks. Blood and visceral adipose tissue, liver tissue and skeletal muscle were examined. Treatment with TA reduced body weight gain, weights of white adipose tissue (WAT) (including epididymal, perirenal, mesenteric WAT and visceral fat), and hepatic triacylglycerol content significantly without affecting food intake in diet-induced diabetic mice. TA effectively prevented HF diet-induced increases in the levels of blood glucose, insulin, leptin and HOMA-IR index (*p* < 0.001, *p* < 0.05, *p* < 0.05, *p* < 0.01, respectively) and attenuated insulin resistance. Treatment with TA, adipocytes in the visceral depots showed a reduction in size. TA effectively significantly increased the protein contents of phosphorylation of AMPK-α (Thr172) both in liver and adipose tissue. It is shown that TA exhibits hypolipidemic effect in HF-fed mice by decreasing gene expressions of fatty acid synthesis, including acyl-coenzyme A: diacylglycerol acyltransferase (DGAT) 2, which catalyzes the final step in the synthesis of triglycerides, and antidiabetic properties occurred as a result of decreased hepatic glucose production via phosphenolpyruvate carboxykinase (PEPCK) down- regulation, improved insulin sensitization and TA (at 1.0 g/kg dose) decreased expression of hepatic and adipose 11-β-hydroxysteroid dehydroxygenase (11β-HSD1) gene, which contributed in attenuating diabetic state. Futhermore, TA at doses of 0.5 and 1.0 g/kg had serum lipid-lowering action characterized by the inhibition of DGAT 1 expression. Thus, amelioration of diabetic and dyslipidemic state by TA in HF-fed mice occurred by regulation of PEPCK, DGAT2 and AMPK phosphorylation.

## Abbreviations

AMPK: AMP-activated protein kinase
aP2: adipocyte fatty acid binding protein
ATGL: adipose triglyceride lipase
BA: 6-benzylaminopurine
BAT: brown adipose tissue
CON: control
CPT-1: carnitine palmitoyl transferase I
DGAT: acyl-coenzyme A: diacylglycerol acyltransferase
EWAT: epididymal white adipose tissue
FAS: fatty acid synthase
FFA: free fatty acid
HF: high-fat control
HOMA-IR: homeostasis model assessment for insulin resistance
MS: Murashige and Skoog
MWAT: mesenteric white adipose tissue
NAA: α-naphthaleneacetic acid
OA: oleanolic acid
PPARγ: peroxisome proliferator-activated receptor γ
PPARs: peroxisomal proliferator-activated receptors
Rosi: rosiglitazone
RWAT: retroperitoneal white adipose tissue
SREBP-1: sterol regulatory element binding protein 1
TA: cell suspension culture of *Eriobotrya japonica*
TC: total cholesterol
TG: triglyceride
WAT: white adipose tissue

## 1. Introduction

Diabetes mellitus and its related metabolic diseases, including type 2 diabetes, dyslipidemia, obesity, and cardiovascular complications, represent a major health problem in the industrialized world. Type 2 diabetes, which accounts for more than 90–95% of all diabetes, is majorly characterized by insulin resistance [1]. Diabetes mellitus is characterized by hyperglycemia that involves abnormalities in both insulin secretion and action at peripheral tissues, resulting in reducing insulin sensistivity at skeletal muscle, adipose and liver tissue. Both genetic and environmental factors play an important role in Type 2 diabetes. Of particular importance may be proportion of fat in the diet. High-fat (HF) diet are well-known to increase body weight, body fat and induce insulin resistance in rodent models. HF diet can also increase liver fat levels quite rapidly (within days) and before significant increases in peripheral fat deposition occur [2]. Such rapid liver fat accumulation is associated with hepatic insulin resistance [2].

The dried leaf of loquat, *Eriobotrya japonica* Lindl. (Rosaceae), is a well known Traditional Chinese Medicine for relieving cough and vomiting. The leaves of loquat are also used in the treatment of diabetes mellitus [3,4]. The reported bioactive components of loquat include flavonoids [5], phenolics [6], amygdalin [7], triterpenic acids [8], and carotenoids [9]. Loquat, which consists of a large amount of pentacyclic triterpenes, would exert many biological activities. The isomeric pentacyclic oleanolic acid, ursolic acid and maslinic acid are the predominant triterpenoids found in loquat leaves [10,11,12,13]. These triterpenes possess many pharmaceutical effects such as hepatoprotective [14] and anti-diabetes actions [12,15]. Tormentic acid could modulate cardiovascular abnormalities [16]. Corosolic acid was reported to exert anti-diabetic activity [17] and ameliorate obesity and fatty liver in KK-Ay mice [18]. Maslinic acid is reported to exert hypoglycemic activity by decreasing hepatic glucose production [19]. Ursolic acid exerts anti-diabetic effects in streptozotocin (STZ)-induced mice [20] and could improve glycemic control and lipid profiles in rodent models [21,22]. Ursolic acid has been identified as a novel PPARα agonist and regulator of hepatic lipid metabolism [23].

Plant cell cultures have been successfully applied to produce large quantities of secondary metabolites from many plants. It is reported that callus tissue culture of *E. japonica* was able to produce large amounts of triterpenes [24]. The optimum culture conditions and the approach for producing large quantities of triterpenes in cell culture are important. Recently, suspension culture was considered that a key progress to achieving commercial scale production [25]. The liquid medium allows the close contact with the tissues, which stimulates and facilitates the uptake of nutrients and hormones [26], leading to better cell growth.

**Figure 1:**
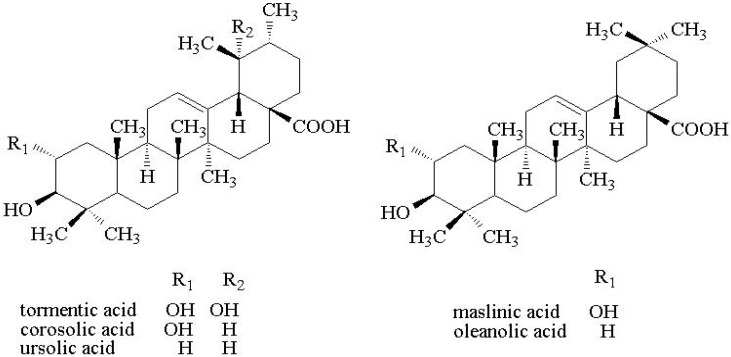
The chemical structures of five triterpenes.

In this study, callus cultures were induced from disinfested seed explants and the best culture conditions on biomass accumulation and triterpenes contents were evaluated by HPLC to determine the best culture conditions. Afterwards, the callus was suspended in different sizes of bioreactor to produce triterpenes successfully on an industrial scale. The loquat cells suspension were extracted and partitioned to obtained fractions rich in triterpenes which were then evaluated for anti-diabetic and anti-hyperlipidemic effects. It was shown that the bioreactor scaled up to 165 L had good yield. The total contents of five triterpenes (Figure 1) were 85.35% and including of tormentic acid, corosolic acid, maslinic acid, oleanolic acid and ursolic acid).

Rosiglitazone, a peroxisome proliferator activated receptor (PPAR)-γ agonist, has been used as an antidiabetic agent in humans [27] and in animals [28,29]. PPARγ is a key regulator of glucose homeostasis [30]. PPAR-γ activators like thiazolidinediones reduce circulating glucose by storing it as fat in adipocytes [31]. A drawback of thiazolidinediones is increase in body weight [32].

Acyl-coenzyme A:diacylglycerol acyltransferase (DGAT), which is the enzyme that catalyzes the final step of triacylglycerol synthesis includes two DGAT isozymes, namely, DGAT1 and DGAT2 [33,34]. DGAT2, which is an enzyme highly expressed in the liver tissue is involved in carrying out the final step in the triglyceride synthesis pathway [35]. DGAT1 activity is distributed in tissues, and is the rate limiting step for the synthesis of triacylglycerol in tissues.

DGAT1-deficient (DGAT1^−/−^) mice have a reduced amount of visceral adipose tissue [36]. They are resistant to high-fat diet-induced obesity [36]. They also showed improvements in glucose, lipid, and energy metabolism and it may partially correlate with altered secretion of adipocytokines such as leptin and adiponectin in mice [37]. Synthetic compounds with strong DGAT1 inhibitory activity have been reported to have anti-obesity [38,39,40] and serum lipid-lowering actions *in vivo* [40,41].

As mentioned above, the components of loquat extract have been shown to ameliorate metabolic syndromes such as diabetes and hyperlipidemia in rodent models. However, the effects of loquat suspension cells (TA) on diabetes and hyperlipidemia *in vivo* remain unknown. 

AMP-activated protein kinase (AMPK) is considered as a therapeutic target for the treatment of diabetes and dyslipidemia [42,43]. Since activation of AMPK results in increased lipid and glucose catabolism [44], the effect of TA on AMPK activity is investigated in mice fed with a HF diet. Phosphorylation of Thr 172 of α subunits is essential for AMPK activity [45]. Recently, adipose triglyceride lipase (ATGL) has been considered as a possible therapeutic target for dyslipidemia and fatty liver [46]. ATGL is responsible for triacyl glycerol hydrolase activity in cells that control the rate-limiting step of lipolysis in many insulin sensitive tissues. ATGL exhibits high specificity for triglyceride to hydrolyze into diglyceride and free fatty acid [47]. As one of the possible mechanisms of action, this study also examined its effect on the expression of genes involved in antidiabetes, lipogenesis and triglyceride lipase in the liver tissue, including diacyl glycerol acyltransferase 1 (DGAT1), diacyl glycerol acyltransferase 2 (DGAT2), 11beta hydroxysteroid dehydrogenase 1 (11beta HSD1), sterol regulatory element binding protein-1c (SREBP-1c), phosphoenol pyruvate caboxykinase (PEPCK) and ATGL.

## 2. Results and Discussion

### 2.1. Body Weight, Body Weight Gain, Food Intake and Tissue Weight

**Figure 2:**
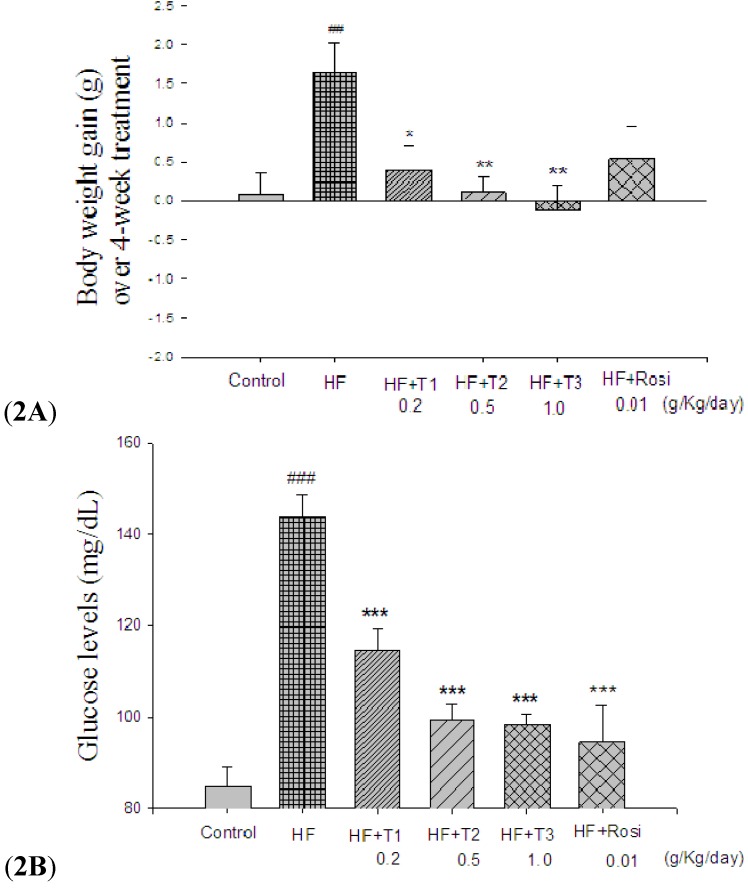
Effect of cell suspension culture of *Eriobotrya japonica* (TA) on (**A**) Body weight gain over 4-week treatment and (**B**) blood glucose levels at week 12. Blood samples were collected from the retro-orbital sinus of fasting mice and the level of glucose was measured by the glucose oxidase method. Mice were fed with 45% high-fat diet (HF) or low-fat diet (CON) for 12 weeks. After 8 weeks, the HF mice were treated with vehicle (water; p.o.), or TA, or rosiglitazone (p.o.) accompanied with HF diet for 4 weeks. All values are means ± S.E. (n=9).^ ##^* p* < 0.01, ^###^* p* < 0.001 compared with the control (CON) group; * *p* < 0.05, ** *p* < 0.01, *** *p* < 0.001 compared with the high-fat + vehicle (distilled water) (HF) group by ANOVA. T1, T2 and T3: cell suspension culture of *Eriobotrya japonica* (T1: 0.2, T2: 0.5 and T3: 1.0 g/kg bodyweight); Rosi: rosiglitazone (0.01 g/kg body weight).

All group mice started with similar mean body weights at the beginning of the study (19.6 ± 0.4 g). At week 12, mice feeding high-fat diet caused significant body weight (Table 1) and weight gain (Figure 2A) compared with mice fed with control diet (*p* < 0.05, *p* < 0.01, respectively). There is no significant difference in the body weight between the TA-treated HF group and vehicle-treated HF group. All the TA-treated groups showed a significant reduction in body weight gain over 4 weeks treatment compared with the HF group (Figure 2A). No significant difference in the 4-week cumulative food intake (kcal) was observed in all the TA- and Rosi- treated groups compared with the HF group (Table 1). At week 12, the weights of absolute adipose tissue (epididymal, mesenteric, retroperitoneal WAT and visceral fat) were markedly greater in the HF group than in the CON group (epididymal WAT 166.1%, mesenteric WAT 58.1%, retroperitoneal WAT 269.7% and visceral fat 120.4%) (*p* < 0.001, *p* < 0.001, *p* < 0.001, *p* < 0.001, respectively). T1, T2 and T3: cell suspension culture of *Eriobotrya japonica (*T1: 0.2, T2: 0.5 and T3: 1.0 g/kg bodyweight); Rosi: rosiglitazone (0.01 g/kg body weight).

**Table 1:** Effects of cell suspension culture of *Eriobotrya japonica* on absolute tissue weight, liver lipids and blood profiles in high-fat-fed mice. All values are means ± S.E (n = 9). ^# ^*p* < 0.05, ^### ^*p* < 0.001 compared with the control (CON) group; * *p* < 0.05, ** *p* < 0.01, *** *p* < 0.001 compared with the high-fat + vehicle (distilled water) (HF) group. Mice were fed with 45% high-fat diet (HF) or low-fat diet (CON) for 12 weeks. After 8 weeks, the HF mice were treated with vehicle (water), or TA, or rosiglitazone accompanied with HF diet for 4 weeks. T1, T2, T3, cell suspension culture of *Eriobotrya japonica.* T1, T2 and T3: cell suspension culture of *Eriobotrya japonica (*T1: 0.2, T2: 0.5 and T3: 1.0 g/kg bodyweight); Rosi: rosiglitazone (0.01 g/kg body weight). BAT, brown adipose tissue; EWAT, epididymal white adipose tissue; RWAT, retroperioneal white adipose tissue; MWAT, mesenteric white adipose tissue; EWAT+ RWAT, visceral fat; FFA, plasma free fatty acid; TC, total cholesterol; TG, triglyceride.

Parameter	CON	HF	HF+T1	HF+T2	HF+T3	HF+Rosi
			0.2 ^a^	0.5 ^a^	1.0 ^a^	0.01 ^a^
Absolute tissue weight (g)						
EWAT	0.513 ± 0.037	1.365 ± 0.134 ^###^	0.859 ± 0.135 *	0.838 ± 0.170 *	0.827 ± 0.138 *	0.801 ± 0.083 *
MWAT	0.375 ± 0.020	0.592 ± 0.041 ^###^	0.451 ± 0.041 *	0.424 ± 0.038 *	0.438 ± 0.042 *	0.383 ± 0.031 **
RWAT	0.142 ± 0.020	0.525 ± 0.050 ^###^	0.301 ± 0.059 *	0.304 ± 0.076 *	0.315 ± 0.043 *	0.255 ± 0.045 **
Visceral fat	0.888 ± 0.053	1.957 ± 0.173 ^###^	1.310 ± 0.180 *	1.262 ± 0.203 *	1.231 ± 0.165 **	1.184 ± 0.073 **
BAT	0.081 ± 0.005	0.092 ± 0.006	0.082 ± 0.007	0.079 ± 0.007	0.077 ± 0.007	0.126 ± 0.006 ***
Liver	0.890 ± 0.032	0.928 ± 0.020	0.917 ± 0.042	0.899 ± 0.040	0.875 ± 0.050	0.893 ± 0.033
Spleen	0.085 ± 0.006	0.091 ± 0.003	0.087 ± 0.003	0.078 ± 0.004	0.082 ± 0.005	0.079 ± 0.002
final bodyweight	26.56 ± 0.74	30.44 ± 1.02^#^	28.80 ± 1.08	28.10 ± 1.17	27.11 ± 1.42	28.27 ± 1.25
4-week cumulative food intake (kcal/mouse)	293.71 ± 9.94	326.50 ± 13.87	307.91 ± 7.56	306.30 ± 12.32	300.58 ± 7.31	304.62 ± 7.41
Liver lipids						
total lipid (mg/g)	56.6 ± 2.3	92.1 ± 5.6 ^###^	78.4 ± 6.1	66.22 ± 5.3 **	63.1 ± 4.2 **	76.3 ± 5.1
Triacylglycerol (μmol/g)	30.7 ± 3.9	74.3 ± 8.2 ^###^	63.8 ± 5.9	48.5 ± 5.1 ***	43.6 ± 5.6 ***	65.6 ± 7.7
Blood profiles						
FFA (meq/L)	1.563 ± 0.083	2.262 ± 0.093 ^#^	2.182 ± 0.213	1.800 ± 0.136	1.663 ± 0.171 *	1.558 ± 0.091 *
TG (mg/dL)	92.5 ± 5.4	137.9 ± 7.6 ^#^	126.0 ± 9.2	107.3 ± 4.6 *	95.3 ± 12.6 *	102.5 ± 7.3 *
TC (mg/dL)	86.4 ± 4.3	150.4 ± 2.6 ^###^	134.8 ± 7.2	124.5 ± 7.3 *	121.8 ± 7.8 *	113.3 ± 5.2 ***
Leptin (μg/mL)	1.49 ± 0.29	6.35 ± 0.83 ^###^	4.69 ± 1.04*	4.37 ± 0.78 *	2.70 ± 0.36 **	4.36 ± 0.87 *
Insulin (μg/L)	0.579 ± 0.029	1.117 ± 0.040 ^###^	0.836 ± 0.052 *	0.821 ± 0.056 **	0.706 ± 0.090 ***	0.660 ± 0.034 ***
Adiponectin (ng/mL)	2.56 ± 0.23	1.76 ± 0.35 ^#^	1.96 ± 0.31	2.31 ± 0.49 *	2.79 ± 0.35 **	3.05 ± 0.57 **
HOMA-IR index	0.81 ± 0.09	2.64 ± 0.39 ^###^	1.58 ± 0.31 **	1.34 ± 0.21 ***	1.14 ± 0.15 ***	1.03 ± 0.18 ***

^a^ Dose (g/kg/day).

Treatment with T1, T2, T3 and Rosi significantly decreased the weights of absolute epididymal, mesenteric and retroperitoneal WAT compared with the HF group. Treatment with T1, T2, T3 and Rosi significantly decreased the weights of visceral fat compared with the HF group (*p* < 0.05, *p* < 0.05, *p* < 0.01, *p* < 0.01, respectively). No significant difference in the weights of liver and spleen was observed in all the TA- and Rosi- treated groups compared with the HF group (Table 1).

### 2.2. Plasma Glucose Levels and Homeostasis Model Assessment for Insulin Resistance (HOMA-IR)

At the beginning of the study, all of mice started with similar levels. At week 12, the glucose levels of the HF group were significantly greater than the CON group by +69.9% (*p* < 0.001). Treatment with T1, T2, T3 and Rosi showed a significant reduction in plasma glucose compared with the HF group (*p* < 0.001, *p* < 0.001, *p* < 0.001, *p* < 0.001, respectively) (Figure 2B). The homeostasis model assessment for insulin resistance (HOMA-IR) was used to calculate insulin resistance, according to the following formula: (milligrams of glucose per deciliter × microunits of insulin per milliliter)/405. Higher numbers indicate greater insulin resistance. At week 12, the levels of HOMA-IR were significantly greater in the HF group than in the CON group. After treatment, T1-, T2-, T3- and Rosi-treated groups showed a significant reduction in HOMA-IR compared with the HF group (Table 1). The data show that rosiglitazone and TA lower glucose by insulin sensitizing, therefore, insulin levels in the groups of mice treated with rosiglitazone and TA showed reduction of insulin as a result of insulin utilization.

### 2.3. Plasma Lipids

As time past, the hypercholesterolemic phenomenon was evident for the HF diet. At week 12, the levels of TC, TG, and FFA were 74.1%, 49.1% and 44.7% greater in the HF group than in the CON group (*p* < 0.001, *p* < 0.05, *p* < 0.05, respectively) (Table 1). Treatment with T2, T3 and Rosi suppressed the HF diet-induced increases in the concentrations of TG (*p* < 0.05, *p* < 0.05, *p* < 0.05, respectively). T2, T3 and Rosi suppressed the HF diet-induced increases in the concentrations of TC by 17.2%, 19.0%, 37.1%, respectively. Treatment with T3 and Rosi suppressed the high-fat diet-induced increases in the concentrations of FFA (*p* < 0.05, *p* < 0.05, respectively) (Table 1).

### 2.4. Leptin, Adiponectin and Insulin Concentration

As shown in Table 1, at week 12, the concentrations of leptin and insulin were greater in the HF group than in the CON group by 76.5%, 92.9%, respectively, whereas the concentrations of adiponectin were lower in the HF group than in the CON group. T1-, T2-, T3- and Rosi-treated groups significantly decreased leptin levels (*p* < 0.05, *p* < 0.05, *p* < 0.01, *p* < 0.05, respectively), whereas T2-, T3 and Rosi- treated groups increased adiponectin levels compared with the HF group (*p* < 0.05, *p* < 0.01, *p* < 0.01, respectively). T1, T2-, T3- and Rosi-treated groups significantly decreased the levels of insulin compared with the HF group (*p* < 0.05, *p* < 0.01, *p* < 0.001, *p* < 0.001, respectively) (Table 1).

### 2.5. Liver Lipids

The liver total lipids and triacylglycerol concentrations were respectively greater in the HF group than in the CON group (Table 1). Treatment with T2 and T3 significantly suppressed the HF diet-induced increase in the liver total lipids and triacylglycerol concentrations (Table 1).

### 2.6. Epididymal WAT Histology

**Figure 3:**
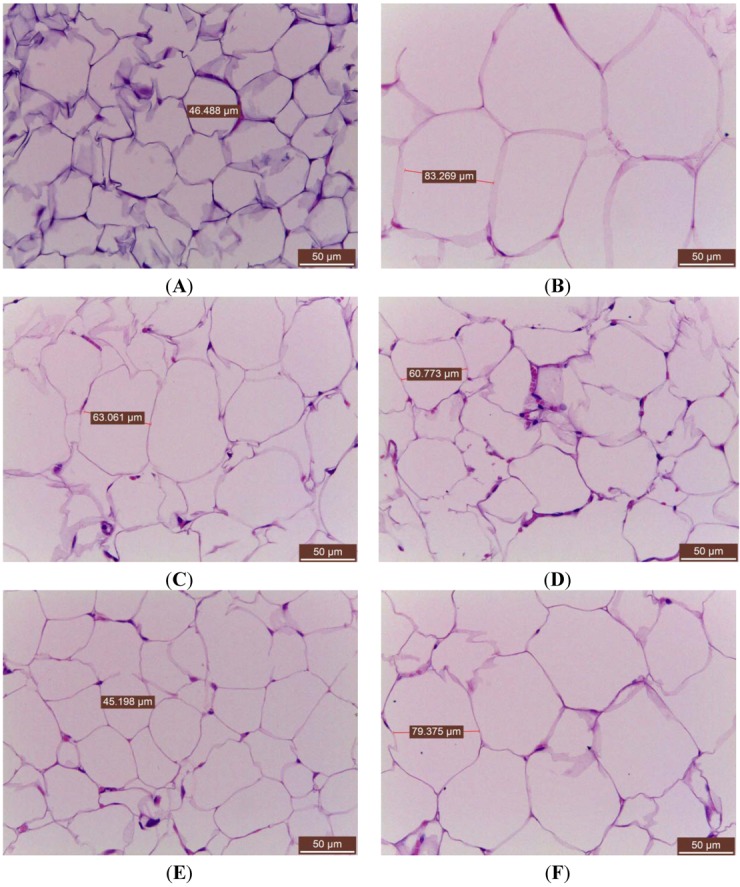
Histology of the epididymal white adipose tissue (WAT) of mice in the (**A**) Low-fat (LF); (**B**) High-fat (HF); (**C**) HF+T1; (**D**) HF+T2; (**E**) HF+T3; or (**F**) HF+Rosi groups. Each presented is typical and representative of nine mice. Magnification: 10 (ocular) × 40 (object lens). T1: 0.2, T2: 0.5 and T3: 1.0 g/kg bodyweight cell suspension culture of *Eriobotrya japonica*; Rosi: rosiglitazone (0.01 g/kg body weight).

Feeding the HF diet induced hypertrophy of the adipocytes (Figure 3B) compared with the CON group (Figure 3A) in epididymal WAT. Following treatment with T1, T2 and T3 decreased the hypertrophy compared with the HF group (Figure 3C–E). The results obtained from the other mice similar to those shown in Figure 3.

### 2.7. Expressions of Apo CIII, DGAT1, DGAT2, PEPCK, 11β-HSD1, ATGL, SREBP1c and Carnitine Palmitoyl Transferase I (CPT-1) in Liver Tissue

**Figure 4:**
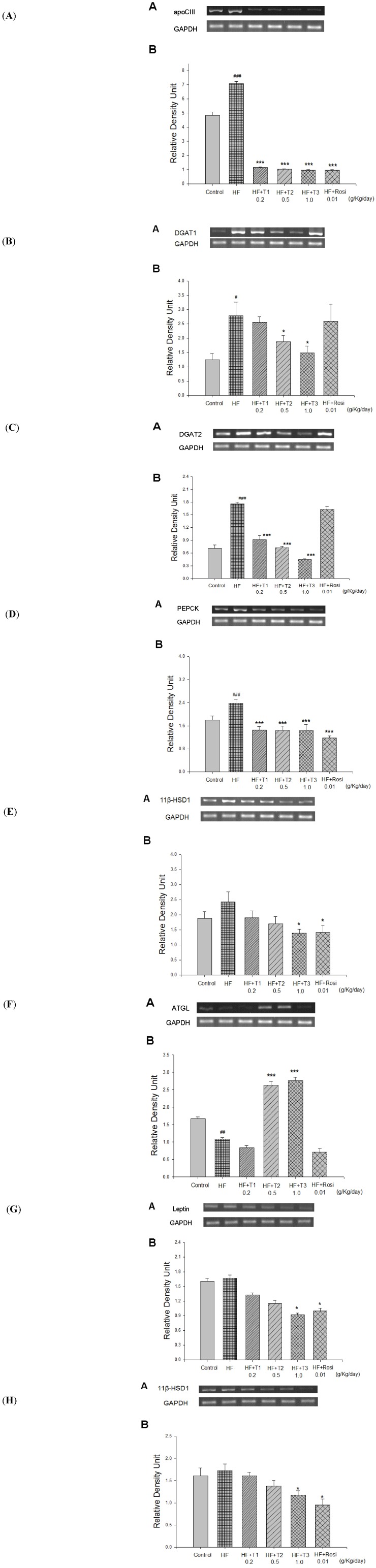
Semiquantative RT-PCR analysis for (**A**) apo C-III, (B) DGAT1, (**C**) DGAT2, (**D**) PEPCK, (**E**) 11β-HSD1 and (**F**) ATGL mRNA expression in liver tissue; (**G**) leptin, and (**H**) 11β-HSD1 mRNA expression in adipose tissue of the mice by oral gavage cell suspension culture of *Eriobotrya japonica* for 4 weeks. All values are means ± S.E. (n = 9). ^# ^*p* < 0.05, ^## ^*p* < 0.01, ^### ^*p* < 0.001 compared with the control (CON) group; * p < 0.05, ** *p* < 0.01, *** p < 0.001 compared with the high-fat + vehicle (distilled water) (HF) group. Total RNA (1μg) isolated from tissue was reverse transcripted by MMLV-RT, 10μL of RT products was used as templates for PCR. Signals were quantitated by image analysis; each value was normalized by GAPDH. T1, T2, T3, cell suspension culture of *Eriobotrya japonica*.

As shown Figure 4 and Table 2, at week 12, the mRNA levels of apo CIII, DGAT1, DGAT2 and PEPCK were higher in the HF group than in the CON group by 46.3%, 122.5%, 147.1%, 32.2%, respectively (*p* < 0.001, *p* < 0.05, *p* < 0.001, *p* < 0.001, respectively), whereas there was no significant difference in 11β-HSD1 and SREBP1c expression of mRNA in the HF group compared with the CON group.

Following treatment, the T1-, T2-, T3- and Rosi- treated groups significantly decreased the mRNA level of apo CIII (*p* < 0.001, *p* < 0.001, *p* < 0.001, *p* < 0.001, respectively). Following treatment, the DGAT1 mRNA level was lower in T2- and T3-treated groups than in the HF group (*p* < 0.05, *p* < 0.05, respectively). T1, T2 and T3 significantly decreased the mRNA level of DGAT2 (*p* < 0.001, *p* < 0.001, *p* < 0.001, respectively). The T1, T2-, T3- and Rosi- treated significantly decreased the mRNA level of PEPCK (*p* < 0.001, *p* < 0.001, *p* < 0.001, *p* < 0.001, respectively). T3 and Rosi significantly decreased the mRNA level of 11β-HSD1 in liver tissue (*p* < 0.05, *p* < 0.05, respectively). At week 12, the mRNA levels of ATGL were lower in the HF group than in the CON group. T2 and T3 significantly increased the mRNA level of ATGL in liver tissue (*p* < 0.001, *p* < 0.001, respectively). T2 and T3 significantly increased the mRNA level of CPT1a in liver tissue (*p* < 0.05, *p* < 0.05, respectively) (Table 2).

### 2.8. Expressions of Leptin, 11β-HSD1 and aP2 in White Adipose tissue

As shown Figure 4 and Table 2, at week 12, there was no significant difference in leptin, 11β-HSD1 and aP2 expression of mRNA in the HF group compared with the CON group. Following treatment, all the T1-, T2- and T3-treated groups decraesed the mRNA level of aP2 expression (*p* < 0.05, *p* < 0.05, *p* < 0.001, respectively). T3 and Rosi significantly decreased the mRNA level of leptin (*p* < 0.05, *p* < 0.05, respectively). T3 and Rosi significantly decreased the mRNA level of 11β-HSD1 in adipose tissue (*p* < 0.05, *p* < 0.05, respectively).

### 2.9. GLUT4 Gene Expression in Skeletal Muscle

At week 12, the skeletal muscular GLUT4 mRNA expressions in the HF group were lower than in the CON group (*p* < 0.05). After treatment, the mRNA expression of GLUT4 was greater in T3- and Rosi-treated groups than in the HF group (Table 2).

**Table 2:** Effects of cell suspension culture of *Eriobotrya japonica* on semiquantative RT-PCR analysis for mRNA expression in liver and white adipose tissue in high-fat-fed mice. All values are means ± S.E. (n = 9). ^# ^*p* < 0.05 compared with the control (CON) group; * *p* < 0.05, *** *p* < 0.001 compared with the high-fat (HF) + vehicle (distilled water) group. Mice were fed with 45% high-fat diet (HF) or low-fat diet (CON) for 12 weeks. After 8 weeks, the HF mice were treated with vehicle (water), or TA, or rosiglitazone accompanied with HF diet for 4 weeks. Total RNA (1 μg) isolated from tissue was reverse transcripted by MMLV-RT, 10 μL of RT products were used as templates for PCR. Signals were quantitated by image analysis; each value was normalized by GAPDH. T1, T2, T3, cell suspension culture of *Eriobotrya japonica.* T1, T2 and T3: cell suspension culture of *Eriobotrya japonica (*T1: 0.2, T2: 0.5 and T3: 1.0 g/kg bodyweight); Rosi: rosiglitazone (0.01 g/kg body weight).

Parameter	CON	HF	HF+T1	HF+T2	HF+T3	HF+Rosi
			0.2 ^a^	0.5 ^a^	1.0 ^a^	0.01 ^a^
Liver						
SREBP-1c	1.117 ± 0.057	1.257 ± 0.096	1.186 ± 0.130	1.046 ± 0.057	1.125 ± 0.062	0.945 ± 0.065
CPT1a	1.438 ± 0.136	1.317 ± 0.165	1.946 ± 0.169	2.052 ± 0.209 *	2.057 ± 0.152 *	1.980 ± 0.432
White Adipose tissue						
PPARγ	1.179 ± 0.093	1.056 ± 0.052	1.078 ± 0.027	1.067 ± 0.113	1.151 ± 0.142	1.410 ± 0.127 *
aP2	1.117 ± 0.050	1.256 ± 0.147	0.958 ± 0.073 *	0.952 ± 0.046 *	0.798 ± 0.045 ***	0.984 ± 0.049
Skeletal muscle						
Glut4	1.441 ± 0.083	1.140 ± 0.064 ^#^	1.188 ± 0.075	1.412 ± 0.099	1.542 ± 0.173 *	1.538 ± 0.053 *

^a^ Dose (g/kg/day).

### 2.10. The Phospho-AMPK (Thr172) Protein Contents in White Adipose and Liver Tissue

**Figure 5:**
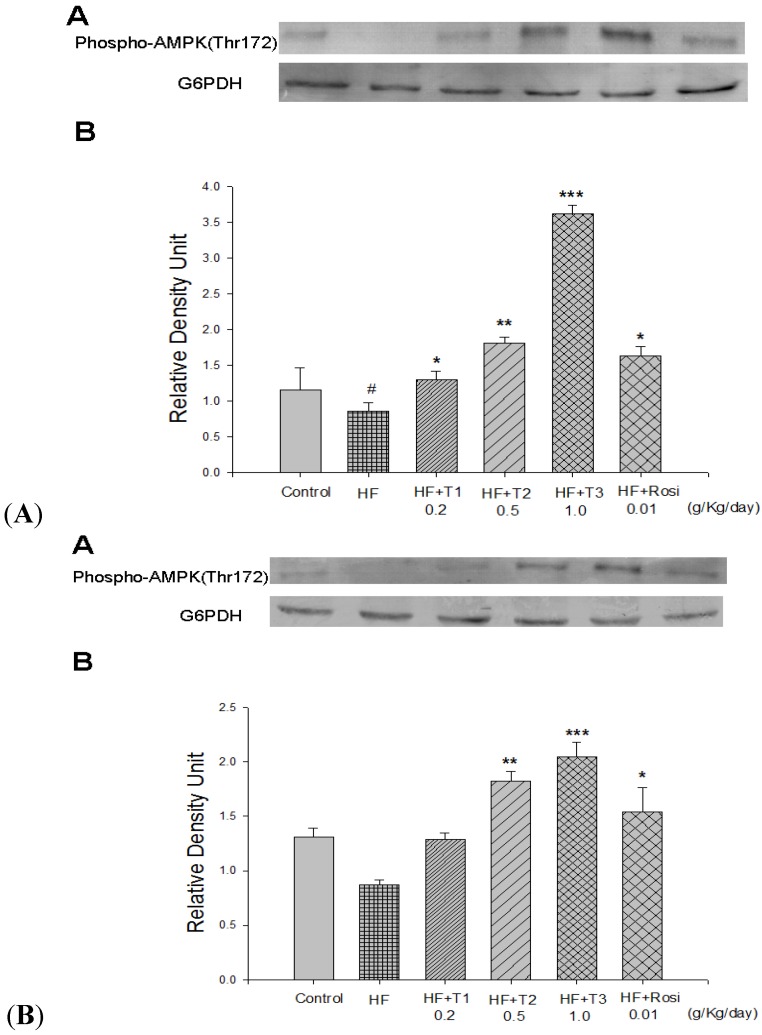
The phospho-AMPK (Thr172) protein contents in liver and white adipose tissue of the mice by oral gavage cell suspension culture of *Eriobotrya japonica* for 4 weeks. Protein was separated by 12% SDS-PAGE detected by Western blot. All values are means ± S.E. (n = 9). ^#^* p* < 0.05 compared with the control (CON) group; * p < 0.05, ** p < 0.01, *** p < 0.001 compared with the high-fat (HF) + vehicle (distilled water) group by ANOVA. T1, T2, T3, cell suspension culture of *Eriobotrya japonica.*

At week 12, the contents of hepatic phospho-AMPK protein were lower in the HF group than in the CON group (*p* < 0.05). There was no significant difference of phosphp-AMPK protein content in white adipose tissue in the HF group compared with the CON group. After treatment, the contents of phospho-AMPK protein increased in the T1-, T2-, T3- and Rosi-treated groups compared with the HF group in liver tissue (*p* < 0.05, *p* < 0.01, *p* < 0.001, *p* < 0.05, respectively) (Figure 5A). Following treatment, the contents of phospho-AMPK protein increased in the T2, T3 and Rosi-treated groups compared with the HF group in adipose tissue (*p* < 0.01, *p* < 0.001, *p* < 0.05, respectively) (Figure 5B).

### 2.11. Oral Glucose Tolerance Test

**Figure 6:**
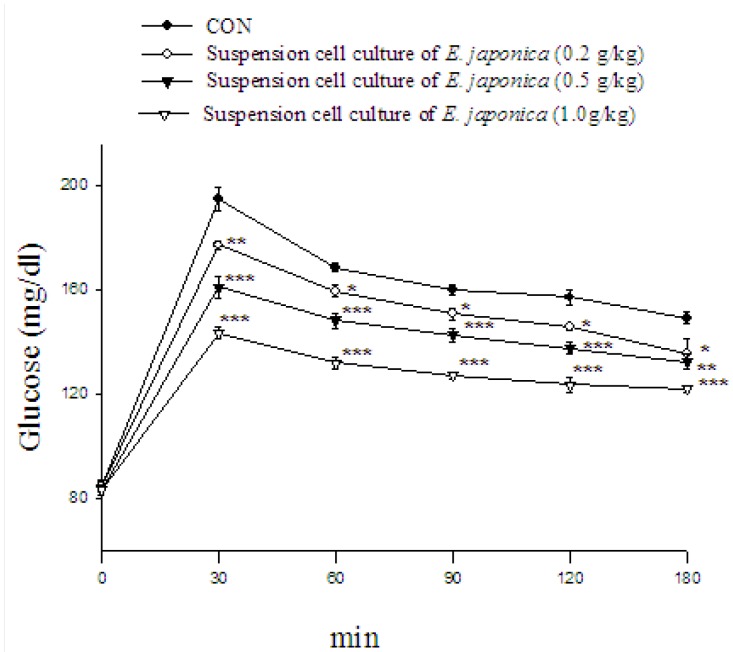
Effects of cell suspension culture of *Eriobotrya japonica* on oral glucose tolerance in normal mice. Animals in all groups received oral glucose 30 min after the extract administration. Blood samples were collected and centrifuged at 3,000 rpm for 10 min. Each point is the mean ± S.E. of 5 separate mice. * *p* < 0.05, ** *p* < 0.01, *** *p* < 0.001 significantly different compared with the control group in the same time by ANOVA.

The effect of cell suspension culture of loquat on OGTT is shown in Figure 6. In the mice treated with 0.2 g/kg, 0.5, 1.0 g/kg significantly decreased blood glucose levels at 30, 60, 90, 120 and 180 min glucose-loading when compared with the control.

### 2.12. Discussion

Although previous studies in our laboratory have demonstrated the effects of the treatment with extract of *E. japonica*, improving insulin resistance, several biochemical and physiological parameters in diet-induced diabetic mice [48], the mechanism of action by which this plant suspension culture exerts its beneficial effects has remained unknown. In this way, the primary objective of the present study was to investigate the mechanism of antidiabetic effect of TA-mediated glucose and lipid lowering in a diabetic and dyslipidemic model, HF-fed mice. The second objective was to examine if anti-hyperlipidemic effects of TA occur via additional mechanism not present in the rosiglitazone treated group. The C57BL/6J mice when fed a HF diet develop severe obesity, hyperglycemia, hyperlipidemia and hyperinsulinemia [49]. Therefore, HF-fed mice model was chosen to address both the antidiabetic and lipid-lowering properties of TA.

The present study demonstrated that TA is effective to improve insulin resistance and dyslipidemia in a mouse model of Type 2 diabetes. Mice treated with TA showed a significant decrease in blood glucose levels. Lower insulin levels were also seen in all the TA-treated mice as compared to the vehicle-treated HF mice. A lower HOMA-IR indicates higher insulin sensitivity [50]. A significant decrease in HOMA-IR was also seen in the TA-administered mice. This could be to the lowered insulin and glucose levels as HOMA-IR reflects the glucose output and insulin secretion.

The first objective of this study was to examine the mechanism of antidiabetic effect of TA and compare with the marketed drug, rosiglitazone, which lowers plasma glucose primarily by insulin sensitization. PEPCK has been proposed to be a key rate-limiting enzyme of gluconeogenesis [51]. Since PPAR-γ agonists are known to cause glucose lowering via inhibiting hepatic glucose production through down-regulation of PEPCK [52], the glucose lowering of TA was examined. The data presented clearly show that TA caused glucose lowering both by inhibiting hepatic glucose production via PEPCK down-regulation, and by insulin sensitization.

The present study demonstrated the antihyperglycemic effect of TA with hepatic PEPCK mRNA suppression in mice fed with a high-fat diet. Previous studies indicated that the hypoglycemic effect was principally attributed to peripheral glucose uptake and hepatic gluconeogenesis suppression [53]. To our knowledge, TA contains bio-constituents involved in the stimulation of peripheral glucose uptake and hepatic gluconeogenesis suppression. Therefore, this suggests that the anti-hyperglycemic effects of TA may also include both the increases of GLUT4 expression and the suppression of hepatic PEPCK expression.

Chronic hyperinsulinemia resulted from compensatory effect of insulin resistance can lead to the degeneration and failure of pancreas beta-cells [50]. In addition, it can also desensitize insulin’s action, thereby causing the regulation of gluconeogenesis and PEPCK expression in obese or type 2 diabetic subjects [52]. High-fat diet is now considered to be pathological factor causing obesity and peripheral insulin resistance [49]. Previous studies also showed that high-fat diet consumption can upregulate PEPCK expression in mice. In the present study, the PEPCK expression increased to a level under a condition of HF diet. Following TA treatment, the PEPCK expression restored to a level lower than the CON group. Since HF diet consumption is related to a decrease in insulin sensitivity, a promising role of the TA as an anti-type 2 diabetes agent is expected and supported by the inhibitory effect of TA on PEPCK expression in HF-fed C57B6J mice. Collectively, it is expected that TA could ameliorate diabetic hyperglycemia by enhancing peripheral glucose uptake and suppressing hepatic glucose over-production.

Moreover, AMPK activation is known to decrease hepatic glucose production and reduce expressions of PEPCK in the liver, thus resulting in reduced glucose levels [51]. In this study, PEPCK expressions were significantly decreased in the liver of all TA- and rosi-treated mice. This could be due to increased contents of phospho-AMPK. This might also indicate that TA has the ability to improve hyperglycemia through TA-stimulated AMPK activities in gluconeogenesis. Therefore, it is possible that TA down-regulated the PEPCK expression, thereby decreasing the plasma glucose level through hepatic AMPK activation.

Another mechanism that could play a role in antidiabetic effect of TA was also investigated. 11β-HSD1 is an enzyme that converts an inactive stress hormone, dehydrocorticosterone (cortisone in humans) into active hormone, corticosterone (cortisol in humans), and 11β-HSD1 knockout mice [54] are protected from developing insulin resistance on high fat diet. Moreover, selective inhibition of 11β-HSD1 has been shown to improve hepatic insulin sensitivity in hyperglycemic KKAy mice [55]. In obese subjects, increased levels of subcutaneous 11β-HSD1 have been shown to be associated with the onset of type 2 diabetes [56]. Thus, compounds that decrease 11β-HSD1 may impart antidiabetic effects. Since 11β-HSD1 is highly expressed in the liver and adipose tissue, the 11β-HSD1 mRNA was quantitated in the liver and in the epididymal fat. The data presented in Figure 4E and 4H demonstrated that the T3- and Rosi-treated group caused a decrease of 11β-HSD1 mRNA both in the liver and epididymal fat. Therefore, in addition to lowering hepatic PEPCK, the lowered 11β-HSD1 mRNA in the liver also contributes to the insulin sensitizing effect of TA.

The second aim of this study was to investigate the mechanism of anti-hyperlipidemic effect of TA. Following treatment with TA, triglycerides lowering occurred as a result of down-regulation of apolipoprotein C-III (apo-C-III), which is a very low density lipoprotein and has been identified as major determinant serum triglycerides. In the present study, further comfirm TA’s lipid lowering effect via down-regulation of genes involved in lipid synthesis. DGAT1 activity is distributed in tissues, and is the rate limiting step for the synthesis of triacylglycerol in tissues. T2 and T3 caused a decrease in DGAT1 mRNA expression. However, in the liver DGAT2 is much more predominantly expressed compared to DGAT1 in both human and mouse. The lipid lowering efficacy of TA was also caused by down-regulation of another enzyme, DGAT2, which catalyzes the final step in the synthesis of triglycerides [35]. Therefore, the down-regulation of DGAT2 appears to be responsible for the hepatic triglyceride output, which, in turn, contributed to be the lowering of circulating triglycerides. 

Following treatment with TA, the visceral fat pad weights were significantly decreased as compared with the vehicle-treated HF group without affecting food intake. Since visceral obesity is thought to play a major role in metabolic syndrome [57], TA might be useful in the treatment of metabolic syndrome associated with visceral obesity, such as hperlipidemia, insulin resistance and Type 2 diabetes. Moreover, T2 and T3 caused a decrease in DGAT1 expression, attenuated body weight gain in diet-induced mice without affecting food intake which is consistent to reports of other DGAT1 inhibitors in the literature [38,39]. TA also showed improvements in glucose, lipid and energy metabolism and it may partially correlate with altered secretion of adipocytokines such as leptin and adiponectin in mice. In this study, we provide the first report that TA has body weight gain-reducing and serum lipid-lowering action characterized by the inhibition of triacylglycerol synthesis in diet-induced mice.

It is known to activation of AMPK may in turn increase ATGL expression and decrease intracellular lipid droplet accumulation [58]. In this study, we showed that TA caused AMPK phosphorylation and increased ATGL expression, which could help for triglyceride to hydrolyze. These data agree with those of Gaidhu et al, who reported that AICAR induces AMPK activation, which promotes energy dissipation through induction of ATGL [58]. Triglyceride hydrolysis resulted in the release of free fatty acids, which were shown to cause insulin resistance. However, TA enhanced AMPK phosphorylation, which in turn increased fatty acid transport to mitochondria for β-oxidation. Carnitine palmitoyl transferase I (CPT-1) is the rate-limiting enzyme for mitochondrial fatty acid oxidation, permitting their entry into the mitochondria for fatty acid oxidation [59]. In addition, it is noteworthy that the CPT-1 mRNA level was significantly higher in the T2- and T3-treated group than in the HF group, thus leading to the increased β-oxidation. These results suggest that such changes also suppressed triacylglyerol accumulation in the liver. Our findings showed that TA may lower lipids and improve insulin sensitivity. Moreover, TA may decrease body weight gain and visceral fat content, and these effects are associated with increased AMPK phosphorylation and ATGL in high-fat-fed mice.

One of the findings of this study is that treatment of mice with TA enhanced adiponectin while decreasing leptin levels. An increase in the concentration of adiponectin will beneficial for insulin sensitizing. TA can provide a unique therapeutic advantage involved in the regulation of adipocyte function to improve insulin sensitivity. In this study, blood leptin levels were elevated by a HF diet and were positively correlated with the increase of visceral fat weight; then after treatment with TA significantly reduced leptin concentrations and mRNA levels. This along with reduction in adipose visceral fat mass is agreement with others reports of adipocyte production and secretion of leptin is reported to be positively corrected to adipose tissue mass [60,61].

It is demonstrated that the treatment of rat adipocytes with globular domain of adiponectin increased glucose uptake and AMPK activation [62]. Adiponectin activates AMPK in the liver, increasing glucose utilization and fatty acid oxidation, and inhibiting glucose production in the liver [63]. Minokoshi *et al.* [64] demonstrated that leptin activated AMPK, and the activation is strongly associated with the enhancement of fatty acid oxidation and suppression of triacylglycerol accumulation. This activation is also performed in adipocytes to prevent excess lipid accumulation in them [64]. It is noteworthy and a novel finding of the present study that the treatment with TA markedly increased the phosphorylation of AMPK. Based on the reports of Wu *et al.* [62] and Minokoshi *et al.* [64], the AMPK phosphorylation by TA may be linked to adiponectin and /or leptin secretion and gene expression. There are two possibilities that TA could directly activate AMPK, or increase plasma adiponectin and decrease leptin concentration by inducing AMPK activation. 

Moreover, aP2 deficiency was reported to protect mice with dietary or genetic obesity from the development of insulin resistance, hyperglycemia and hypertriglyceridemia [65,66]. TA results in decreased adipose tissue aP2 expression, thus has a favorable impact on multiple components of metabolic syndrome by protecting from diet-induced obesity, insulin resistance, Type 2 diabetes and fatty liver disease. In conclusion, we have demonstrated that TA affected adipocytokine (adiponectin and leptin) secretion and adipocytes specific gene (aP2), and AMPK phosphorylation would be associated with these changes. Our findings provide a biochemical basis for the use of TA which can also have important implications for controlling diabetes and hyperlipidemia. 

## 3. Experimental

### 3.1. Callus Culture Establishment

Since cell suspension culture materials are offered by our team partners Ho *et al.* and professor Wu. The following Section 3.1, Section 3.2, Section 3.4 procedures are according to Ho *et al.* [68,69]. Briefly, seeds of *E. japonica* Lindl were provided by Mr. Chen-I Chen, Department of Bio-industry and Agribusiness Administration in Taiwan. The seed surfaces were sterilized in 70% (v/v) ethanol, followed by 1% (w/v) sodium hypochlorite supplemented with Tween 20, and rinsed three times with sterile distilled water. The seeds were then placed on the Murashige and Skoog (MS) basal medium [67] containing 3% (w/v) sucrose. After one month, the leaves were harvested and weighed separately from the culture medium. The tissue was then sliced into 2- to 3-mm slices and transplanted into MS medium supplemented with 2.5 mg/L BA and 1 mg/L NAA for callus induction. The calli were all grown at 25 ± 2 °C in the dark.

### 3.2. Callus Induction from Leaves of E. japonica Lindl and Initiation of Suspension Cultures

The 20-day-old callus (about 1.5 g) induction from leaves of *E. japonica* Lindl was transferred to a Erlenmeyer flask containing 100 mL MS medium supplemented with BA, NAA, and 3% (w/v) sucrose. The calli were then grown at 25 ± 2 °C in the dark for 18 days. All fresh calli were collected and then dried at 60 °C for 48 h to determine triterpene content. Callus cultures were subcultured every 20 days. Suspension cultures were established by inoculating the 3 g of 20-day-old callus masses in 1-L Erlenmeyer flasks containing 400 mL liquid MS medium. These cultures were incubates on a rotary shaker at 120 rpm. The temperature was maintained at 25 ± 2 °C in the dark.

### 3.3. Bioreactor Method

The biomass method is initiated by 0.5 L culture liquid is added to 4.5 L fresh culture to culture, then 4.5 L culture liquid is transferred to 35 L bioreactor. 4.5 L cell culture liquid is added to 25.5 L fresh culture liquid, then 25 L liquid (about 3500 g) under 35 L bioreactor for 10 days. Finally, 25 L culture liquid is added to 120 L culture liquid (containing MS, 2.5 mg/l BA, 1 mg/NAA and 3% sucrose) under 165 bioreactor to culture at 24~26 °C for 18 days.

### 3.4. Determination of Triterpene Content

Triterpenes were extracted from 1 g of dried cell with 20 mL of 95% ethanol at 70 °C for 8 h, three times. The combined ethanolic extracts were filtered and the filtrate was concentrated under reduced pressure (centrifugal evaporator CVE 3100, Eyela, Japan). About 50 mg of the condensed extract was re-dissolved in 10 mL of methanol/water (85:15) under 20-min sonication to ensure the complete extraction of triterpenes, and filtered. Then, 20 μL of the filtered extract was subjected to HPLC triplicate. The triterpene content was determined by HPLC. In brief, HPLC was performed on a Shimadzu 10A system equipped with one pump (LC-10AT Shimadzu, Kyoto, Japan) and an RI spectrophotometric detector (RID-10A), Shimadzu, Japan). The mobile phase (methanol: 0.15% aqueous acetic acid = 85: 15) was pumped at a flow rate of 0.5 mL min^−1^ with a HyPURITY C-18, <phi> 4.6 × 250 mm HPLC column. The cycle time of analysis was 40 min. The total contents of five triterpenes were 85.35% (tormentic acid 44.30%, corosolic acid 19.50%, maslinic acid 14.65%, oleanolic acid 1.60% and ursolic acid 5.30%, respectively).

### 3.5. Animals and Experimental Design

All procedures were approved by the Institutional Animal Care and Use Committee of Central Taiwan University of Science and Technology. Male C57BL/6J mice (4–5 weeks old) were obtained from the National Laboratory Animal Breeding and Research Center, National Science Council. The animals were housed in an air-conditioned room at 22 ± 3 °C with 12 h of light and tap water *ad libitum*. After a 1-week acclimation period, the mice were divided randomly into two groups. The control (CON) group (n = 9) was fed low-fat diet (Diet 12450B, Research Diets, Inc., New Brunswick, NJ, USA), whereas the experimental group was fed a 45% high-fat diet (Diet 12451, Research Diets, Inc.) for 12 weeks. The low-fat diet was composed of protein 20%, carbohydrate 70% and fat 10%, whereas high-fat diet was composed of protein 20%, carbohydrate 35% and fat 45% (of total energy, % kcal). After 8-week diet-induction period, the high-fat treated mice were randomly subdivided into 5 groups. Loquat cell suspension culture (including 0.2, 0.5, 1.0 g/kg/day) or rosiglitazone (Rosi; 1% methylcellulose 10 mg/kg body weight, obtained from GlaxoSmithKline Product No: BRL49653 C)) were administrated through oral gavage 1 time per day from 9 to 12 week of the experiment, and the mice were still on the high-fat diet, while the CON and high-fat control (HF) mice were treated with vehicle only. The body weight was measured weekly throughout the study. The dietary design lasted for 12 weeks. The compositions of the experimental diets are shown in Table 3.

At the end of experiment, the mice were sacrificed by exsanguinations, and the weights of the tissues were measured. The liver and white adipose tissues (WATs) (including epididymal, mesenteric and retroperitoneal WAT) were dissected according to the defined anatomical landmarks. Visceral fat was defined as the sum of epididymal and retroperioneal WAT. Tissues were then immediately frozen using liquid nitrogen and kept at −80 °C until use.

Blood sample was allowed to clot at room temperature for 5 min. Plasma samples were collected by centrifugation at 1,600 × g for 15 min at 4 °C. The separation of the plasma was finished within 30 min. Aliquots of the supernatant were obtained for insulin, leptin, total cholesterol (TC), TG and FFA assay. The plasma was immediately frozen at −80 °C until use.

**Table 3:** Composition of the high- and low- fat diets (kcal).

Ingredient	Low-fat	High-fat
Casein	800	800
L-Cystine	12	12
Corn starch	1,260	291
Maltodextrin 10	140	400
Sucrose	1,400	691
Cellulose, BW200	0	0
Soybean Oil	225	225
Lard	180	1,598
Mineral Mix S10026	0	0
Dicalcium carbonate	0	0
Calcium carbonate	0	0
Potassium citrate, 1H_2_O	0	0
Vitamin Mix V10001	40	40
Choline bitartrate	0	0
FD&C Yellow Dye #5	0	
FD&C Red Dye #40		0
FD&C Blue Dye #1		
Total	4,057	4,057

### 3.6. Food Intake and Body Weight Assay

Firstly, the pellet food was weighed then placed in the cage food container. After 24 h, the remaining food was weighed. The difference represented the daily food intake. The animal weight and food weight were measured using an electronic scale. Unconsumed pellet HF food was discarded each day and fresh pellet high-fat diet was provided to ensure consistent food quality throughout the study. The HF food was stored at 4 °C.

### 3.7. Blood Parameters Assay

Blood samples were collected from the retro-orbital sinus of fasting mice and the level of glucose was measured by the glucose oxidase method (Model 1500; Sidekick Glucose Analyzer; YSI Incorporated, Yellow Springs, OH, USA). The concentrations of triglyceride (TG), total cholesterol (TC) and free fatty acid (FFA) were measured using commercial assay kits according to the manufacturer’s directions (Triglycerides-E test, Cholesterol-E test and FFA-C test, Wako Pure Chemicals, Osaka, Japan).

### 3.8. Adipocytokine Levels Assay

The levels of insulin and leptin were measured by ELISA using a commercial assay kit according to manufacturer’s directions (mouse insulin ELISA kit, Sibayagi, Gunma, Japan and mouse leptin ELISA kit, Morinaga, Yokohama, Japan).

### 3.9. Histology

Small pieces of epididymal WAT were fixed with formalin (200 g/kg) neutral buffered solution and embedded in paraffin. Sections (8 µm) were cut and stained with hematoxylin and eosin. For microscopic examination, a microscope (Leica, DM2500) was used, and the images were taken using a Leica Digital camera (DFC-425-C) at 10 (ocular) × 40 (object lens) magnification.

### 3.10. Measurement of Hepatic Lipids

Hepatic lipids were extracted using a previously described protocol [48,70]. For the hepatic lipid extraction, the 0.375 g liver samples were homogenized with 1 mL distilled water for 5 min. Finally, the dried pellet was resuspended in 0.5 mL ethanol and analysed using a triglycerides kit as used for serum lipids.

### 3.11. Isolation of RNA and Relative Quantization of mRNA Indicating Gene Expression

Total RNA from the epididymal WAT, skeletal muscle and liver was isolated with a Trizol Reagent (Molecular Research Center, Inc., Cincinnati, OH, USA) according to the manufacturer’s directions. The integrity of the extracted total RNA was examined by 2% agarose gel electrophoresis, and the RNA concentration was determined by the ultraviolet (UV) light absorbency at 260 nm and 280 nm (Spectrophotometer U-2800A, Hitachi). The quality of the RNA was confirmed by ethidium bromide staining of 18S and 28S ribosomal RNA after electrophoresis on 2% agarose gel containing 6% formaldehyde.

Total RNA (1 μg) was reverse transcribed to cDNA in a reaction mixture containing buffer, 2.5 mM dNTP (Gibco-BRL, Grand Island, NY, USA), 1 mM of the oligo (dT) primer, 50 mM dithiothreitol, 40 U Rnase inhibitor (Gibco-BRL, Grand Island, NY), and 5μL Moloney murine leukemia virus reverse transcriptase (Epicentre, Madison, WI, USA) at 37 °C for 1 h, and then heated at 90 °C for 5 min to terminate the reaction. The polymerase chain reaction (PCR) was performed in a final 25μL containing 1U Blend Taq™ -Plus (TOYOBO, Osaka, Japan), 1 μL of the RT first-strand cDNA product, 10 μΜ of each forward (F) and reverse (R) primer, 75 mM Tris-HCl (pH 8.3) containing 1 mg/L Tween 20, 2.5 mM dNTP and 2 mM MgCl_2_. Preliminary experiments were carried out with various cycles to determine the nonsaturating conditions of the PCR amplification for all the genes studied. The primers are shown in Table 4. The products were run on 2% agarose gels and stained with ethidium bromide. The relative density of the band was evaluated using AlphaDigiDoc 1201 software (Alpha Innotech, Co. San Leandro, CA, USA). All the measured PCR products were normalized to the amount of cDNA of GAPDH in each sample.

**Table 4:** Primers used in this study.

Gene	Accession numbers	Forward primer and reverse primer	PCR product (bp)	Annealing temperature (°C)
White adipose tissue				
PPARγ	NM_013124	F: CATGCTTGTGAAGGATGCAAG	190	55
		R: TTCTGAAACCGACAGTACTGACAT		
Leptin	NM_008493	F: GGCATTTTCTTACCTCTGTG	303	55
		R: ACTTTGGATGAACCAATCAG		
aP2	NM_024406	F: TCACCTGGAAGACAGCTCCT	143	50
		R: TGCCTGCCACTTTCCTTGT		
SREBP1c	NM_011480	F: GGCTGTTGTCTACCATAAG	219	55
		R: AGGAAGAAACGTGTCAAGAA		
FAS	NM_007988	F: TGGAAAGATAACTGGGTGAC	240	55
		R: TGCTGTCGTCTGTAGTCTTG		
Liver				
apo C-III	NM_023114.3	F: CAGTTTTATCCCTAGAAGCA	349	47
		R: TCTCACGACTCAATAGCTG		
CPT-1	NM_153679	F: GCAGGAAATTTACCTCTGTG	288	55
		R: ACATGAAGGGTGAAGATGAG		
DGAT1	NM_010046.2	F: ATCTTTGCTCCTACTTTGTGTT	333	50
		R: ATTCCACCAATCTCTGTAGAAC		
DGAT2	NM_026384.3	F: AGTGGCAATGCTATCATCATCGT	149	50
		R: AAGGAATAAGTGGGAACCAGATCA		
11β-HSD1	NM_008288.2	F:AAGCAGAGCAATGGCAGCAT	300	50
		R: GAGCAATCATAGGCTGGGTCA		
PPARα	NM_011144	F: ACCTCTGTTCATGTCAGACC	352	55
		R: ATAACCACAGACCAACCAAG		
FAS	NM_007988	F: TGGAAAGATAACTGGGTGAC	240	50
		R: TGCTGTCGTCTGTAGTCTTG		
SREBP1c	NM_011480	F: GGCTGTTGTCTACCATAAGC	219	50
		R: AGGAAGAAACGTGTCAAGAA		
PEPCK	NM_011044.2	F: CTACAACTTCGGCAAATACC	330	52
		R: TCCAGATACCTGTCGATCTC		
Skeletal muscle				
Glut4	M25482	F: ACTGGCGCTTTCACTGAACT	106	55
		R: CGAGGCAAGGCTAGATTTTG		
GAPDH	NM_031144	F: TGTGTCCGTCGTGGATCTGA	99	55
		R: CCTGCTTCACCACCTTCTTGA		

### 3.12. Western Immunoblotting Analysis of Phospho-AMPK (Thr172) Proteins

Protein extractions and immunoblots for the determination of AMPK phosphorylation were carried out on frozen liver and adipose tissue from mice according to a previous report [71]. Briefly, liver samples (0.1 g) were powdered under liquid nitrogen and homogenized for 20 s in 500 μL buffer containing 20 mM Tris-HCl (pH 7.4 at 4 °C), 2% SDS, 5 mM EDTA, 5 mM EGTA, 1 mM DTT, 100 mM NaF, 2 mM sodium vanadate, 0.5 mM phenylmethylsulfonyl fluoride, 10 μg/mL leupeptin and 10 μL/mL pepstatin. A 40 μg sample of each homogenate was mixed with an equal amount of 2× standard SDS sample loading buffer containing 125 mM Tris-HCl (pH 6.8), 4% SDS, 20% glycerol, 10% β-mercaptoethanol and 0.25% bromophenol blue, and boiled for 10 min before electrophoresis.

Proteins were separated by 12% SDS-PAGE according to the method of Laemmli [72] and transferred by electroblotting onto PolyScreen PVDF transfer membrane (NEN) using semi-dry transfer cell (Bio-Rad) according to the manufacturer’s manual. The membrane was then treated sequentially with blocking solution (phosphate-buffered saline (PBS) containing 5% non-fat skim milk), with appropriate dilution of anti-phospho-AMPKα (Thr 172) antibody (Abcam Inc, Cambridge, MA, USA), and with anti-(G6PD) G6PD (glucose 6 phosphate dehydrogenase antibody; Abcam Inc, USA) conjugated to peroxidase (Zymed Inc, South San Francisco, CA, USA). Finally, the membrane was soaked in a chromogen/substrate solution (TMB single solution; Zymed) for color development.

### 3.13. Oral Glucose Tolerance test (OGTT)

The normal mice (n = 5) were fasted for 15–18 h but were allowed access to 0.2 g/kg, 0.5 g/kg, 1.0 g/kg cell suspension culture of loquat, or an equivalent amount of normal saline was given orally 30 min before an oral glucose load (1 g/kg body weight). Blood samples were collected at the time of the glucose administration (0) and every 30 min until 3 h after glucose administration to determine the levels of glucose.

### 3.14. Statistical Analysis

Data were expressed as mean ± S.E. values. Whenever possible, data were subjected to analysis of variance, followed by Dunnett’s multiple range test, using SPSS software (SPSS Inc., Chicago, IL, USA). *p* < 0.05 was considered to be statistically significant.

## 4. Conclusions

It is clearly that treatment with TA decreased levels of triglycerides and glucose in HF-fed mice. It is worth nothing that TA exhibits antidiabetic properties occurred as a result of increased hepatic AMPK phosphorylation, whereas inhibition of PEPCK mRNA, thus resulting in decreased hepatic glucose output, improved insulin sensitization. Moreover, TA decreased the expression of hepatic DGAT 2, which, in turn, contributed to be the lowering of circulating triglycerides.
